# Perceived Social and Emotional Support from Co-Workers and Supervisors and Occupational Stress in Emergency Physicians: A Pre-Pandemic National Cross-Sectional Study from Slovenia

**DOI:** 10.3390/jcm15197488

**Published:** 2026-09-26

**Authors:** Meta Košir, Miha Košir

**Affiliations:** 1 Psihoterapija Meta Košir (Private Practice), 1000 Ljubljana, Slovenia; 2 Department of Family Medicine, Faculty of Medicine, University of Ljubljana, Vrazov Trg 2, 1000 Ljubljana, Slovenia; 3 Emergency Medical Service (SNMP), Community Health Centre Ljubljana, Zaloška Cesta 7, 1000 Ljubljana, Slovenia

**Keywords:** occupational stress, emergency medicine, social support, supervisor support, peer support, burnout

## Abstract

**Background/Objectives:** Emergency physicians report high levels of occupational stress, but its organisational determinants remain under-investigated, particularly in small national systems. This exploratory, pre-pandemic study examined occupational stress among emergency medicine specialists in Slovenia, hypothesising that lower perceived support from supervisors and from co-workers would be associated with greater stress. **Methods:** All 86 emergency medicine specialists registered in Slovenia were surveyed in March–April 2019, and 45 participated (52.3%). Stress and support were measured with the Brief Job Stress Questionnaire and analysed with correlation coefficients, *t*-tests and Mann–Whitney tests, with multivariable regression as a sensitivity analysis. **Results:** Support was perceived as lowest from supervisors and highest from partners, family and friends. Lower perceived supervisor support was associated with poor interpersonal relationships at work (r = 0.568), poor job fit, a poor work environment, lower job control and total stress (r = 0.488); lower co-worker support was only associated with poor job fit and work perceived as not worth doing. Neither was associated with the intrinsic demands of the work. After adjustment for sex, age and tenure, supervisor support (β = 0.49) and female sex (β = 0.34) remained independently associated with total stress (adjusted R^2^ = 0.30). **Conclusions:** The hypothesis was supported for supervisor support but only partly for co-worker support. The small sample, multiple comparisons and cross-sectional design make these findings exploratory and specific to the pre-pandemic period. As perceived support is organisationally modifiable, leadership and peer support are identified as targets for future intervention research, and multinational studies in comparable emergency systems are warranted.

## 1. Introduction

Since the pre-COVID-19 period, emergency medicine has been consistently identified as one of the medical specialties most exposed to occupational stress; in the French SESMAT study, conducted in 2008, emergency physicians accumulated more stress factors than colleagues in other specialties, including time pressure, work–family conflict and demanding working conditions [1]. A 2020 meta-analysis found that approximately 40% of emergency physicians reach high levels of emotional exhaustion and depersonalisation, exceeding rates in most other specialties [2], and a systematic review published in the same year reports a burnout prevalence ranging from 25.4% to 71.4% [3]. A more recent meta-analysis, published in 2023, of 29 studies covering 16,619 emergency department healthcare workers estimated overall burnout at 43%, with 35% at high risk, and documented higher rates during the pandemic than before it [4]. A 2025 systematic review confirms that stress in this setting extends beyond the workplace, affecting mental health and private life alike [5]. National cross-sectional surveys in other health systems report comparable burdens among physicians [6].

Several features of emergency medicine explain this concentration of risk. Emergency physicians are regularly exposed to work-related traumatic events, which are associated with post-traumatic symptoms and psychological distress [7]; a 2026 meta-analysis of 150 studies confirms that this exposure translates into a substantial burden, with a pooled prevalence of probable post-traumatic stress disorder among healthcare workers of 15.5% before the pandemic, rising to 24.8% during it [8]. Shift and night work impose an additional and cumulative burden: in a 2014 longitudinal study of US emergency physicians, night work substantially reduced satisfaction with the specialty and prompted a considerable proportion to consider leaving the field [9], and a 2025 evaluation of a night-shift buy-out programme found that more than 80% of physicians who reduced their night shifts regarded this as very important or critical for remaining in the job [10]. A recent national survey of Swiss emergency physicians found that more than half met at least one criterion for burnout and that 40.6% had considered leaving emergency medicine because of working conditions [11], illustrating that these pressures translate directly into workforce attrition.

Slovenia provides a distinctive setting for studying these questions. It is a country of approximately two million inhabitants operating a Franco-German model of emergency care, in which prehospital emergency services are physician-staffed and emergency departments are distributed across both hospital and non-hospital sites—community health centres; emergency medicine was recognised as an independent specialty only in 2007 [12]. The workforce implications are already apparent: a 2022 national survey of Slovenian medical students and pre-residency doctors found that only 4% gave a clearly positive response regarding a career in emergency medicine, and identified shift length, workload and collegiality as decisive factors [13].

Organisational factors are central to contemporary models of occupational stress. According to the job demands–resources model, first formulated in 2007, the harmful effects of high job demands depend in part on the job resources available, among which social support from supervisors and co-workers is one of the most important [14], a proposition that has been retained and extended in the most recent formulation of the theory [15] and that continues to be supported empirically: a 2026 meta-analysis of 31 studies of intensive care nurses found social support to be inversely associated with burnout (r = −0.40) and work–family conflict positively associated with it (r = 0.49) [16]. Workplace social support is associated with lower levels of experienced stressors and strain [17], and the buffering hypothesis holds that support protects the individual against the harmful effects of stress [18], a mechanism that continues to be invoked in current accounts of burnout and engagement [15,16]. Evidence from emergency medicine, collected before the pandemic, is consistent with this [19], and the distinction between sources of support matters, because supervisor behaviour is organisationally modifiable [20,21].

Despite this literature, empirical data on the relationship between perceived support and occupational stress in emergency medicine specialists remain limited. In the region, a Croatian study, published in 2011, comparing 79 emergency medical service physicians with 81 health centre physicians found that the former experienced more frequent uncontrollable work situations, greater work–family conflict and less favourable relationships with co-workers [22], but it used a bespoke stress scale and compared work settings rather than isolating sources of support.

To our knowledge, no study has examined perceived supervisor and co-worker support in relation to occupational stress among emergency physicians in Slovenia. The aim of this study was therefore to investigate factors associated with occupational stress among emergency medicine specialists in Slovenia, focusing on perceived social and emotional support from supervisors and co-workers, and on sociodemographic and work-related characteristics. We hypothesised that lower perceived social and emotional support from supervisors and from co-workers would be associated with greater occupational stress; differences by sex, age and job tenure were examined as exploratory questions without directional hypotheses.

## 2. Materials and Methods

### 2.1. Study Design and Setting

This was a national cross-sectional survey of emergency medicine specialists in Slovenia, conducted between March and April 2019, that is, before the COVID-19 pandemic, and forming part of a broader research project on occupational stress in this population [23]. The study was conducted in the Slovenian emergency medical system described above, comprising physician-staffed prehospital emergency units of community health centres and hospital-based emergency departments [12]. Reporting follows the STROBE recommendations for cross-sectional studies [24], which remain the reference standard for observational research [25].

### 2.2. Participants and Recruitment

The sampling frame comprised all 86 physicians registered as emergency medicine specialists in the Republic of Slovenia on 3 March 2019, according to publicly available data from the Medical Chamber of Slovenia. A census approach was used, with all physicians in the sampling frame considered for recruitment. Eligibility required completed specialist training in emergency medicine and current clinical work in an emergency medical service or emergency department. Because no central register permitting direct contact with individual emergency medicine specialists was available, recruitment was conducted through the heads of all emergency departments and emergency medical units within community health centres in Slovenia. The heads of these units were asked to forward the study invitation and survey link to eligible emergency medicine specialists employed at their institution. Participation was voluntary and anonymous, and no incentives were offered. The survey was open between 6 March 2019 and 20 April 2019. A single invitation was issued and no reminders were sent. The response rate was defined as the number of respondents providing valid answers divided by the 86 specialists in the sampling frame.

In addition to the study instrument, the questionnaire collected four sociodemographic and occupational characteristics: sex (man, woman), age (in five-year bands from 30–35 years to 60 years or more), marital status (in a relationship or cohabiting, married, widowed, divorced, single) and years of work in emergency medical services (in five-year bands from 0–5 years to 31 years or more). No other personal or workplace identifiers were collected in order to preserve anonymity in a small and professionally identifiable population.

### 2.3. Instrument

Occupational stress and perceived support were measured with the Brief Job Stress Questionnaire (BJSQ) [26], first developed in 2000, a generic instrument developed for the assessment of psychosocial working conditions across occupational sectors and used nationwide in the Japanese Stress Check Program [27,28]; its continued use is documented in a 2023 systematic review of 145 observational studies [29]. The BJSQ comprises 57 items rated on four-point scales, covering job stressors (17 items), psychological and physical stress responses (29 items) and buffering factors including social support at work [28]. A systematic review of 145 observational studies published between 2003 and 2021 found that the subscales used most frequently were quantitative workload, job control and support from supervisors and co-workers and concluded that these subscales demonstrate construct validity as representative job stressors within the job demand–control framework [29]; these are the subscales on which the present analysis principally relies. The present analysis used the 17 job-stressor items, the 29 stress-response items and the 9 social support items. Items were reverse-scored where appropriate so that higher values consistently indicate a less favourable state, that is, greater stress or lower support. Following the scoring approach documented in that review [29], subscales were computed for quantitative workload, qualitative (mental) demands, physical demands, low job control, infrequent use of skills at work, poor interpersonal relationships, poor physical work environment, job fit and work perceived as worth doing, together with a total job-stressor score (range 17–68); and, from the support section, support from supervisors, from co-workers and from partners, family and friends, together with a total support score (range 9–36).

The BJSQ was administered in Slovenian. A professional translation of the published English version of the instrument was used; the Slovenian version has not undergone formal cross-cultural validation according to established procedures for forward–backward translation and psychometric testing [30], procedures that remain the standard approach in contemporary instrument adaptation studies [31], and no validated Slovenian version of the BJSQ has been published for any occupational group. A validation study within this population is not feasible, as the entire national workforce of emergency medicine specialists numbered 86 at the time of the survey. We therefore assessed internal consistency within our own sample, which was acceptable to high (job-stressor section α = 0.740 after reverse scoring; stress-response section α = 0.927; social and emotional support section α = 0.810) and comparable to values reported for the original instrument in large working populations [28]. Internal consistency, however, does not establish measurement equivalence with the original instrument, and this is acknowledged as a limitation that bears on every comparison with studies using validated versions of the instrument.

### 2.4. Statistical Analysis

Data were analysed in IBM SPSS Statistics version 22.0 (IBM Corp., Armonk, NY, USA) [32]. All analyses were conducted on complete cases: every respondent included had answered all items of the BJSQ, so no values were missing on the study variables and no imputation was performed. Descriptive statistics are reported as frequencies and percentages for categorical variables and as means and standard deviations for continuous variables. Normality of distribution was assessed with the Shapiro–Wilk test [33], which is appropriate for samples of *n* < 50 and remains the most widely applied test of normality in applied research [34]. Associations were assessed with Pearson’s coefficient (r) [35] where normality was confirmed for both variables and with Spearman’s coefficient (ρ) [36] otherwise; both coefficients remain in routine use in current occupational-health research, for example in a 2026 meta-analysis of correlates of burnout [16], and current methodological guidance continues to address the choice between them [34]. Group differences were tested with independent-samples *t*-tests [37] where normality was confirmed, with Levene’s test [38] used to select the appropriate variant and with Mann–Whitney tests [34,39] otherwise. Age and job tenure were dichotomised at the sample median for group comparisons. The significance level was set at α = 0.05.

The primary analyses were bivariate. With 45 participants, a multivariable model including the two support variables together with sex, age and tenure approaches the lower bound of acceptable observations per predictor, so bivariate associations were treated as the principal results. Because occupational stress may nevertheless be confounded by sex, age and job tenure, a multivariable linear regression was performed as a sensitivity analysis to establish whether the bivariate associations with perceived support persisted after adjustment (Section 3.6). The total job-stressor score was the outcome, and lack of supervisor support, lack of co-worker support, sex, age group and tenure group were entered simultaneously as predictors. Unstandardised and standardised coefficients with 95% confidence intervals are reported; residual normality was checked with the Shapiro–Wilk test and multicollinearity with variance inflation factors. Given the sample size, this model is interpreted as a robustness check rather than as an independent test of the hypothesis. Ninety-five per cent confidence intervals for correlation coefficients were obtained by the Fisher z transformation. No correction for multiple testing was applied. This decision follows the principle that adjustment is warranted when particular results are emphasised because of their small *p*-values [40]; here, all associations are reported in full, several *p*-values lie close to the conventional threshold, and the analyses are explicitly presented as exploratory and hypothesis-generating rather than confirmatory. For transparency, the number of tests performed is reported alongside the results, and readers may apply a false-discovery-rate criterion [41], an approach whose application continues to be governed by current guidance on when adjustment is warranted [40] if they wish to interpret the findings under a confirmatory frame.

During the preparation of this manuscript, the authors used a generative artificial intelligence assistant (Anthropic Claude) for the purposes of language editing and assisting with literature searching, and, during revision, for scripting the re-analysis of the primary survey data under the authors’ direction. All references identified with its assistance were independently verified by the authors against primary academic databases, and all statistical results were independently reproduced by the authors in IBM SPSS Statistics. The tool was not involved in the study design, data collection or interpretation of the findings. The authors have reviewed and edited all output and take full responsibility for the content and interpretation presented in this publication.

### 2.5. Ethical Considerations

The study was conducted in accordance with the Declaration of Helsinki and approved by the National Medical Ethics Committee of the Republic of Slovenia (approval no. 0120-466/2017/5; date of approval: 19 September 2017). The introductory page of the anonymous online questionnaire informed respondents of the purpose of the study, the topics covered and the anonymity of all responses and requested their voluntary participation. Proceeding to complete the questionnaire was taken as informed consent. Because the survey was anonymous and no personal data were collected, no signed consent form was obtained, in keeping with the Committee’s approval of the protocol.

## 3. Results

### 3.1. Participant Characteristics

The survey platform recorded 68 accesses, of which 23 were clicks on the invitation without any answers and were discarded. Forty-five specialists provided valid responses, giving a response rate of 52.3% of the national specialist population. All 45 completed the demographic section and the entire BJSQ, so the analyses reported below are based on complete data from all 45 respondents; nine of them discontinued in later sections of the questionnaire that addressed partner relationships and are not used in the present analysis.

Of the 45 participating specialists, 23 were men (51.1%) and 22 were women (48.9%). The age distribution was skewed towards younger specialists: a total of 21 respondents (46.7%) were aged 30–35 years and a further 15 (33.3%) were aged 36–45 years, while only three participants (6.7%) were older than 50 years. Most respondents were married (48.9%) or in a partnership (37.8%). Sixty per cent had up to 10 years of tenure in emergency medical services, with the most frequent tenure category being 6–10 years (37.8%). Participant characteristics are presented in Table 1.

### 3.2. Occupational Stress and Perceived Support

The mean total job-stressor score was 43.60 (SD = 5.67; observed range 31–55 on a possible range of 17–68), somewhat above the scale midpoint. Among individual items, respondents agreed most strongly that they must be very attentive and careful in their work (M = 3.84; SD = 0.42) and that their work requires a high level of knowledge and technical skill (M = 3.71; SD = 0.59). The mean total stress-response score over the preceding month was below the scale midpoint (M = 51.04; SD = 12.26; observed range 31–87 on a possible range of 29–116), indicating a comparatively favourable stress response on average.

Perceived support differed markedly by source. Support was perceived as lowest from supervisors (M = 2.48; SD = 0.68), followed by co-workers (M = 2.01; SD = 0.54), and highest from partners, family and friends (M = 1.36; SD = 0.41), with higher values denoting a greater perceived lack of support. The total lack-of-support score was below the scale midpoint (M = 17.56; SD = 3.75; possible range 9–36).

### 3.3. Support from Supervisors and Co-Workers and Dimensions of Occupational Stress

Twenty correlations were computed (two sources of support × ten stress dimensions). A greater perceived lack of support from supervisors was positively associated with poor interpersonal relationships at work (r = 0.568; 95% CI 0.33–0.74; *p* < 0.001), poor job fit (ρ = 0.447; 0.18–0.66; *p* = 0.002), a poor physical work environment (ρ = 0.401; 0.12–0.62; *p* = 0.006), lower job control (ρ = 0.345; 0.06–0.58; *p* = 0.020), the perception that one’s work is not worth doing (ρ = 0.314; 0.02–0.56; *p* = 0.035) and the total job-stressor score (r = 0.488; 0.23–0.68; *p* = 0.001). A greater perceived lack of co-worker support was associated with the perception that one’s work is not worth doing (ρ = 0.403; 0.12–0.62; *p* = 0.006) and with poor job fit (ρ = 0.320; 0.03–0.56; *p* = 0.032), but not with job control, interpersonal relationships or the total job-stressor score. The confidence intervals are wide, and several of the associations of small-to-moderate magnitude have lower bounds close to zero; they should be interpreted with corresponding caution. Neither source of support was associated with quantitative workload, qualitative (mental) demands or physical demands (Table 2).

### 3.4. Differences by Sex

Men and women did not differ in the general stress response (U = 245.5; *p* = 0.865). Among the dimensions of occupational stress, women reported lower job control (M = 2.91 vs. 2.51; U = 139.5; *p* = 0.008); the difference in the rating of the physical work environment did not reach significance (M = 2.55 vs. 2.00; U = 193.0; *p* = 0.153). Total job-stressor scores were higher among women (M = 45.64; SD = 5.67) than among men (M = 41.65; SD = 5.04; t = −2.494; *p* = 0.017). No other dimension differed by sex.

### 3.5. Differences by Age and Job Tenure

Between younger (30–35 years; *n* = 21) and older respondents (36 years or more; *n* = 24), and between physicians with shorter (up to 10 years; *n* = 27) and longer tenure in emergency medical services (11 years or more; *n* = 18), a difference emerged for a single dimension only: physical demands were rated higher by older respondents (M = 2.67 vs. 2.24; U = 160.0; *p* = 0.018) and by those with longer tenure (M = 2.72 vs. 2.30; U = 152.5; *p* = 0.018); as age and tenure are closely related in this sample, these two findings largely reflect the same respondents. The total job-stressor score and all other dimensions did not differ by age or tenure. The stress-response score tended to be lower among older (*p* = 0.053) and longer-serving respondents (*p* = 0.057), but neither difference reached significance.

### 3.6. Multivariable Sensitivity Analysis

In the multivariable linear regression (Table 3), the five predictors together explained 30% of the variance in the total job-stressor score (adjusted R^2^ = 0.297; F(5, 39) = 4.72; *p* = 0.002). A greater perceived lack of supervisor support remained independently associated with higher total stress after adjustment for co-worker support, sex, age and tenure (b = 4.10 per scale point; 95% CI 1.55–6.65; β = 0.49; *p* = 0.002), as did female sex (b = 3.81; 95% CI 0.78–6.84; β = 0.34; *p* = 0.015). Lack of co-worker support (b = −0.52; 95% CI −3.68 to 2.65; *p* = 0.742), age group and tenure group were not associated with total stress in the adjusted model. A model containing the two support variables only explained 21% of the variance (adjusted R^2^ = 0.206), with the same pattern (supervisor support *p* = 0.002; co-worker support *p* = 0.656). Residuals were normally distributed (Shapiro–Wilk *p* = 0.969) and variance inflation factors were below 3, indicating no problematic multicollinearity. The sensitivity analysis therefore confirms the bivariate finding for supervisor support and suggests that the bivariate associations of co-worker support with individual stress dimensions do not translate into an independent association with total stress.

## 4. Discussion

In this exploratory, pre-pandemic national survey covering more than half of all emergency medicine specialists in Slovenia, perceived support from supervisors and co-workers was associated with multiple dimensions of occupational stress, and supervisors were identified as the weakest source of support while partners, family and friends were the strongest. These findings were consistent with our hypothesis for supervisor support, which was also the only source of support independently associated with total stress in the adjusted model; for co-worker support the hypothesis received only partial support, confined to two dimensions of stress and not extending to total stress. Two features of the pattern of associations are of particular interest, with the caveats set out in Section 4.1 applying throughout.

First, support was not associated with the intrinsic demands of the work, namely quantitative workload, mental demands and physical demands, but rather with its relational and value-related dimensions: the quality of interpersonal relationships, the rating of the work environment, job fit, the sense that one’s work is worth doing, and total stress. This is coherent with the job demands–resources model, in which social support functions as a job resource that does not reduce demands themselves but shapes how burdensome they are experienced to be [14,15], with meta-analytic evidence linking workplace support to lower strain, reported in 1999 [17] and confirmed in a 2026 meta-analysis [16], and with the stress-buffering hypothesis, formulated in 1985 [18]. In emergency medicine, where workload and cognitive demands are largely inherent to the clinical task and cannot be substantially reduced, this distinction may have practical significance: it suggests that support is among the more modifiable levers available to managers, although the correlational design does not establish that changing support would change stress.

Second, our findings align with those reported among 256 Japanese emergency physicians surveyed in 2014, where low supervisor support, low job control and poor job suitability were each associated with burnout [19], and with the Croatian comparison, published in 2011, in which emergency medical service physicians reported less favourable relationships with co-workers than health-centre physicians [22]. This convergence should be read with an important reservation: the Japanese studies used the validated original BJSQ, whereas our study used an unvalidated Slovenian translation, so that similarity of pattern cannot be taken as evidence of measurement equivalence, and differences in magnitude cannot be interpreted. With that reservation, the convergence of studies conducted in markedly different healthcare systems is at least compatible with the view that supervisor support is a cross-culturally relevant correlate of stress in this specialty rather than an artefact of local circumstances.

The finding that perceived support was lowest precisely for supervisors, while its associations with stress were strongest, directs attention to the quality of leadership in emergency care. Because the design is cross-sectional, the direction of causation cannot be determined: a lack of support from leaders may increase stress; physicians under greater strain may perceive less support; or a third factor, such as understaffing, may drive both—and recent methodological work shows that even longitudinal designs can generate spurious causal claims about social support [42]. The wider literature nevertheless indicates that leadership behavior is a plausible target rather than a fixed characteristic: in a multicentre survey of 5416 physicians, those rating their supervisor’s leadership in the highest tertile were 48% less likely to be burned out and reported 66% lower intent to leave than those in the lowest tertile [20], and, in a 2015 study, each one-point increase in a composite leadership score was associated with a 3.3% reduction in the likelihood of burnout [21]. The observation that support from partners, family and friends was rated highest is consistent with the possibility that emergency physicians in this system compensate for a deficit of institutional support with private resources, although this interpretation could not be tested directly.

Differences by sex require cautious interpretation. Men and women did not differ in their general stress response, but women reported lower job control and higher total stress, and female sex remained independently associated with total stress in the adjusted model. This is consistent with a systematic review of 18 studies covering 26,939 emergency physicians from 10 countries, which found that overall burnout rates did not differ between the sexes but that a higher proportion of female emergency physicians reported high emotional exhaustion (69% vs. 57%) and a low sense of personal accomplishment (45% vs. 29%) [43], and with earlier evidence identifying lower perceived job control among women physicians in 2000 [44] and greater difficulty reconciling professional and family demands in 2012 [45]. Because the differences here are confined to specific dimensions and derive from a small sample, they should not be generalised into a claim that women experience more stress overall; they indicate rather that particular features of the work environment may be experienced as more burdensome by women, which would be relevant to the design of any future intervention.

The absence of differences in total stress by age or tenure, as well as the isolated finding of greater physical demands among older and longer-serving physicians, is consistent with the structurally similar exposure of all career stages in emergency medicine, combined with the cumulative physical toll of prolonged shifts and night work [9,10]. Given that a substantial proportion of emergency physicians in comparable systems consider leaving the specialty because of working conditions [11] and that emergency medicine already struggles to attract new entrants in Slovenia [13], this observation bears on workforce sustainability while recognising that the present data predate the pandemic-related changes in working conditions.

### 4.1. Strengths and Limitations

The principal strength of this study is its coverage: 52.3% of the entire national population of emergency medicine specialists participated, so the findings approach a population-level description rather than a convenience sample. The study also has several limitations, each of which is associated with a recognised form of bias.

First, selection and non-response bias. Selection bias arises whenever the analysed sample is conditioned on a common effect of exposure and outcome, a structure formalised in 2004 [46], and non-response is its most common source in survey research. The entire national population of emergency medicine specialists is small, which constrains sample size while making near-complete national coverage attainable; nevertheless, close to half of eligible specialists did not participate. Non-response is rarely random [47]: a 2025 study of healthcare employee well-being surveys found that non-respondents had markedly higher subsequent turnover and lower productivity than respondents, indicating that those under greatest strain are the least likely to answer [48]. If the most burdened emergency physicians in Slovenia were similarly under-represented, the associations reported here would tend to be underestimated rather than inflated, but the direction of any bias cannot be verified. Invitations were distributed through unit heads rather than directly. Only a single invitation was sent, and the sample was skewed towards younger specialists (most respondents were aged 30–45 years and only three were older than 50), so the findings relate primarily to younger members of the specialty and their generalisability to the whole workforce is limited.

Second, limited statistical power and multiplicity. Because Slovenia had only 86 emergency medicine specialists at the time of the survey, even complete participation would have yielded a modest absolute sample; with *n* = 45, statistical power is limited to the detection of moderate-to-large effects, and the absence of significant associations cannot be interpreted as evidence that such associations do not exist. Low power also increases the chance that a significant result overestimates the true effect [49]; this remains a live methodological concern, and current guidance emphasises that underpowered studies miss real effects and that sample-size planning is design-specific rather than formula-specific [50]. Twenty correlations and further group comparisons were tested without adjustment for multiplicity, and several *p*-values lie close to 0.05; under a false-discovery-rate criterion [41] some of the weaker associations, particularly those involving co-worker support, would not survive. Consistent with current guidance on when adjustment is required [40], we therefore present all results as exploratory and refrain from emphasising individual findings on the basis of their *p*-values. This ceiling is a structural property of small national workforces and can be overcome only through multinational collaboration.

Third, measurement bias arising from the instrument. The Slovenian version of the BJSQ used here was a professional translation of the English version and has not been formally validated according to accepted cross-cultural adaptation procedures [30], which remain the standard in current adaptation studies [31]; measurement equivalence with the original instrument therefore cannot be assumed, and comparisons with studies using the validated Japanese version should be made with corresponding caution. This limitation is not unique to our study. A systematic review of BJSQ use excluded several international studies precisely because the validity of the translated instrument had not been established [29], but it constrains the strength of the inferences that can be drawn. In addition, all variables were self-reported through a single instrument at a single point in time, creating the conditions for common-method bias, which can inflate correlations between constructs measured in the same way [51,52]; this concern has been raised for the BJSQ specifically [28].

Fourth, the cross-sectional design precludes causal inference. Cross-sectional studies measure prevalence at a single point in time and cannot distinguish cause from effect [53]; the associations reported are compatible with several causal orderings and with confounding, and no claim about the effect of changing support on stress can be made from these data—a caution reinforced by recent work showing that even longitudinal analyses of social support can generate spurious causal conclusions [42].

Fifth, and very important for interpretation, data were collected in 2019, before the COVID-19 pandemic, which substantially intensified the burden on emergency departments and altered burnout profiles internationally [4]. Because a cross-sectional survey characterises a population only at the time of measurement [53], the secular change introduced by the pandemic is not a peripheral caveat but a boundary on interpretation: a 2026 meta-analysis found that probable post-traumatic stress disorder among healthcare workers rose from 15.5% before the pandemic to 24.8% during it, returning towards pre-pandemic levels only by the third year [8]. The present findings therefore describe the pre-pandemic baseline of a national specialty workforce. They should not be generalised to the current period, and any consideration of their organisational relevance must begin from the recognition that working conditions have changed since the data were collected.

### 4.2. Future Research Directions

Because the data are correlational and pre-pandemic, they do not by themselves justify specific organisational or policy interventions. They do, however, identify candidate targets and testable propositions for future research, which we set out below.

First, the study should be replicated in the post-pandemic period with the same instrument and the same national sampling frame. This would enable direct comparison with the pre-pandemic baseline reported here and establishing whether the pattern of associations, support related to relational and value-related dimensions of stress but not to intrinsic demands, has persisted or changed. Also, the number of emergency medicine specialist in Slovenia has increased since the study in 2019 was performed.

Second, the sample-size ceiling inherent to small national workforces should be addressed through multicentre international studies inviting countries with comparably organised, physician-staffed emergency medical services; such collaboration would provide the statistical power for adequately specified multivariable modelling of the determinants of stress, including adjustment for sex, age and tenure, which the present sample could support only as a sensitivity analysis.

Third, the three practical propositions suggested by our findings warrant testing in intervention research rather than adoption on the strength of these data alone: (i) that structured leadership development for the heads of emergency medical service units and emergency departments would reduce occupational stress, given that supervisor support was perceived as the weakest source and showed the strongest associations with stress, and that leadership behaviour has been linked to physician burnout elsewhere [20,21]; (ii) that structured peer-support programmes at team level would reduce stress, given the weaker but consistent associations with co-worker support; and (iii) that rostering which accounts for tenure and age would mitigate the cumulative physical burden of long-term shift work, as suggested by the greater physical demands reported by longer-serving physicians and by the emerging evidence on schedule flexibility [10]. Each of these is a hypothesis for a prospective or quasi-experimental study, not a recommendation derived from the present results.

Fourth, formal cross-cultural adaptation and validation of the BJSQ in Slovenian, following established forward–backward translation and psychometric procedures [30] and conducted in a larger healthcare population such as hospital physicians or nurses, would establish the measurement basis for its use in this setting and permit sound international comparison; the instrument has already been translated into ten other languages [29], so a Slovenian adaptation would be a natural extension.

## 5. Conclusions

This pre-pandemic national survey aimed to investigate factors associated with occupational stress among emergency medicine specialists in Slovenia, testing the hypothesis that lower perceived social and emotional support from supervisors and co-workers would be associated with greater occupational stress. The results corroborated the hypothesis for supervisor support, which was associated with the relational and value-related dimensions of stress and with total stress, and corroborated it more weakly and less consistently for co-worker support; neither source of support was associated with the intrinsic demands of the work. Supervisors were perceived as the weakest source of support. Women reported lower job control, a poorer rating of the work environment and higher total stress, and physicians with longer tenure reported greater physical demands. Given the small sample, the number of comparisons, the unvalidated translation of the instrument, the cross-sectional design and the pre-pandemic timing of data collection, these findings are exploratory. Perceived support is amenable to organisational change; the results therefore identify leadership and peer support as candidate targets for future intervention research in emergency medicine rather than as a basis for immediate policy change. Multinational studies involving healthcare systems with comparable physician-staffed emergency services are warranted to address the sample-size limitations inherent in research conducted in small countries.

## Figures and Tables

**Table 1:** Characteristics of participating emergency medicine specialists (*n* = 45).

Characteristic	Category	*n*	%
Sex	Men	23	51.1
	Women	22	48.9
Age	30–35 years	21	46.7
	36–40 years	7	15.6
	41–45 years	8	17.8
	46–50 years	6	13.3
	51–55 years	2	4.4
	60 years or more	1	2.2
Marital status	Married	22	48.9
	In a partnership/cohabiting	17	37.8
	Single	5	11.1
	Divorced	1	2.2
Tenure in EMS	0–5 years	10	22.2
	6–10 years	17	37.8
	11–15 years	8	17.8
	16–20 years	8	17.8
	21–25 years	1	2.2
	26–30 years	1	2.2

EMS, emergency medical services. Percentages may not sum to 100.0 owing to rounding. No respondent fell into the 56–60 years category or reported being widowed. For the analysis of group differences, age was dichotomised at 30–35 years (*n* = 21) versus 36 years or more (*n* = 24), and tenure at up to 10 years (*n* = 27) versus 11 years or more (*n* = 18).

**Table 2:** Associations between perceived lack of social and emotional support from supervisors and co-workers and dimensions of occupational stress (BJSQ) (*n* = 45).

Stress Dimension (BJSQ)	Lack of Supervisor Support			Lack of Co-Worker Support		
	Coefficient	95% CI	*p*	Coefficient	95% CI	*p*
Quantitative workload	r = 0.168	−0.13 to 0.44	0.270	ρ = −0.010	−0.30 to 0.28	0.948
Qualitative (mental) demands	ρ = 0.235	−0.06 to 0.49	0.120	ρ = −0.001	−0.29 to 0.29	0.993
Physical demands	ρ = −0.045	−0.33 to 0.25	0.771	ρ = 0.044	−0.25 to 0.33	0.774
Low job control	ρ = 0.345 *	0.06 to 0.58	0.020	ρ = 0.072	−0.23 to 0.36	0.640
Infrequent use of skills at work	ρ = 0.043	−0.25 to 0.33	0.781	ρ = −0.158	−0.43 to 0.14	0.301
Poor interpersonal relationships	r = 0.568 **	0.33 to 0.74	<0.001	ρ = 0.281	−0.01 to 0.53	0.061
Poor physical work environment	ρ = 0.401 **	0.12 to 0.62	0.006	ρ = 0.138	−0.16 to 0.41	0.368
Poor job fit	ρ = 0.447 **	0.18 to 0.65	0.002	ρ = 0.320 *	0.03 to 0.56	0.032
Work perceived as not worth doing	ρ = 0.314 *	0.02 to 0.56	0.035	ρ = 0.403 **	0.12 to 0.62	0.006
Total job-stressor score	r = 0.488 **	0.23 to 0.68	0.001	ρ = 0.161	−0.14 to 0.43	0.291

BJSQ, Brief Job Stress Questionnaire; CI, confidence interval. Coefficients are Pearson’s r where normality was confirmed for both variables and Spearman’s ρ otherwise; 95% CIs were obtained by the Fisher z transformation. * *p* < 0.05; ** *p* < 0.01. Higher lack-of-support values indicate lower perceived support, and higher stress-dimension values greater stress; positive coefficients therefore indicate that less support is associated with more stress. Twenty tests were performed without adjustment for multiplicity (Section 2.4).

**Table 3:** Multivariable linear regression of the total job-stressor score on perceived lack of support, sex, age and tenure (*n* = 45).

Predictor	b (95% CI)	β	*p*	VIF
Lack of supervisor support (per scale point)	4.10 (1.55 to 6.65)	0.49	0.002	1.42
Lack of co-worker support (per scale point)	−0.52 (−3.68 to 2.65)	−0.05	0.742	1.39
Female sex (vs. male)	3.81 (0.78 to 6.84)	0.34	0.015	1.11
Age 36 years or more (vs. 30–35)	0.89 (−3.75 to 5.54)	0.08	0.700	2.62
Tenure 11 years or more (vs. up to 10)	−2.86 (−7.44 to 1.72)	−0.25	0.215	2.45

b, unstandardised regression coefficient; CI, confidence interval; β, standardised coefficient; VIF, variance inflation factor. Model: adjusted R^2^ = 0.297; F(5, 39) = 4.72; *p* = 0.002; intercept = 33.26. Outcome is the total job-stressor score (possible range 17–68). Support scores range from 1 (full support) to 4 (no support).

## Data Availability

The data presented in this study are available on request from the corresponding author due to privacy restrictions: the study population is small and professionally identifiable, and full participant anonymity could not otherwise be guaranteed. The dataset also underlies a separate analysis of the partner-relationship dimension, reported elsewhere.
